# Routine post-operative full blood count assessment is not necessary in elective hip and knee arthroplasty: A prospective cohort study

**DOI:** 10.1016/j.jcot.2025.103099

**Published:** 2025-06-14

**Authors:** David Moore, Henry Turner, Jess Rotaru, Ciara Doran, James Cashman

**Affiliations:** a Royal College of Surgeons in Ireland, 123 St Stephen's Green, Dublin, D02 YN77, Ireland; b National Orthopaedic Hospital Cappagh, Cappagh Rd, Cappoge, Dublin, D11 EV29, Ireland

**Keywords:** Haemoglobin, Full blood count, Total hip arthroplasty, Total knee arthroplasty, Haemorrhage, Transfusion

## Abstract

**Background:**

Hip and knee arthroplasty are among the commonest orthopaedic procedures performed worldwide and can be associated with significant blood loss. Routine haemoglobin sampling increases transfusion rates without an overall reduction in morbidity and mortality, yet providers commonly adopt an absolute numerical value as warranting transfusion post-operatively. Our aim was to establish what proportion of patients had a significant reduction in haemoglobin requiring transfusion thus assessing the necessity of routine post-operative haemoglobin assessment in an inpatient and outpatient patient cohort undergoing total joint replacement.

**Methods:**

We performed a review of prospectively collected data in an institutional database of all primary elective hip and knee arthroplasty cases performed by a single surgeon at an urban tertiary referral centre from 2018 to 2023. We assessed pre-operative and post-operative variables amongst patients to identify predictors for transfusion following surgery. All statistics were performed using Stata release 17.

**Results:**

490 elective primary hip or knee arthroplasty procedures were performed within the six year period of which the mean drop in haemoglobin was 2.1 g/dL (SD 1.0, Range −5.6 to +1) post-operatively. Four patients (0.8 %) had a haemoglobin drop below 8 g/dL and 19 (3.9 %) had a level below 9 g/dL, however only 4 patients (0.8 %) required allogenic blood transfusion. One hundred and six patients (21.9 %) underwent day-case arthroplasty of which there was no re-admissions or complications within 90 days of surgery.

**Conclusion:**

The routine monitoring of haemoglobin following elective TJA is costly and unnecessary whilst not contributing to actionable information. We recommend that patients with a pre-operative level greater than 11 g/dL may not require routine post-operative full blood count as mean drop in haemoglobin of less than 3 g/dL can be expected. We continue to advocate that day-case arthroplasty is safe in appropriately selected patients as defined by ASA grade at anaesthetic pre-assessment.

**Level of evidence:**

Level II – Prospective cohort study.

## Highlights

• Routine haemoglobin sampling increases transfusion rates without an overall reduction in morbidity and mortality.
• 490 elective primary hip or knee arthroplasty procedures were performed within the six year period of which the mean drop in haemoglobin was 2.1 g/dL.
• 0.8 % had a haemoglobin drop below 8 g/dL and 3.9 % had a level below 9 g/dL, only 4 patients (0.8 %) required allogenic blood transfusion.
• 106 (21.9 %) underwent day-case arthroplasty of which there was no re-admissions or complications within 90 days of surgery.
• The routine monitoring of haemoglobin following elective TJA is a costly unnecessary endeavour and does not contribute to actionable information.

## Graphical abstract

**Figure:**
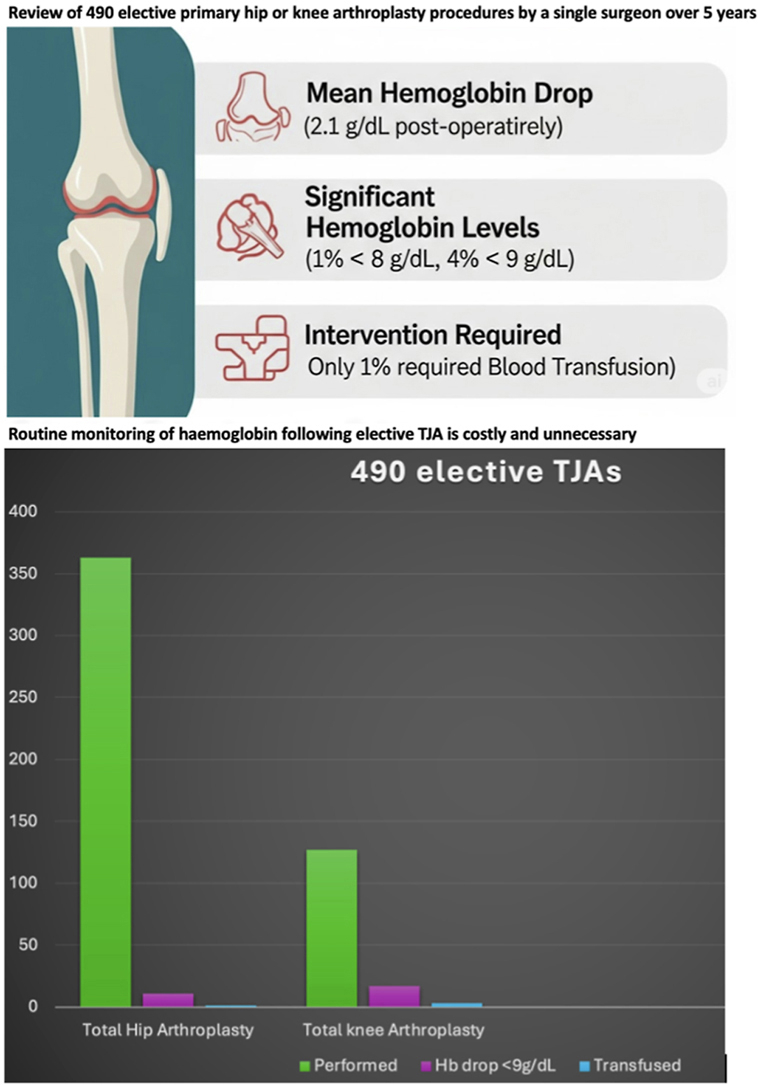

## 1 Background

Hip and knee arthroplasty are among the commonest procedures performed worldwide^1^ and can be associated with significant blood loss. It is common practise to measure serum haemoglobin following surgery to identify anaemia however, blood transfusion following total joint arthroplasty (TJA) is undesirable as it is associated with infection and transfusion reactions.^2^, ^3^, ^4^, ^5^, ^6^, ^7^, ^8^, ^9^ Modern perioperative techniques have reduced the need for transfusion. Previous transfusion rates in elective TJA have been as high as 50 %^10^ but this figure has dropped with advances in the last decade.^1^, ^11^, ^12^, ^13^ The use of hypotensive anaesthesia, shorter operative times and anti-thrombolytic agents have reduced the number of patients whose post-operative haemoglobin values meet the transfusion threshold.

Routine haemoglobin sampling increases transfusion rates without reducing morbidity and mortality, yet providers commonly adopt an absolute numerical value as warranting transfusion in the post-operative setting.^14^ Risk factors for transfusion have been extensively studied with older age, female gender, medical comorbidities and a haemoglobin value of 13.0 g/dL or less increasing ones risk of requiring transfusion post-operatively.^11^, ^13^, ^15^, ^16^ Despite all of this, haemoglobin sampling is still routinely performed without a clear indication.^17^

The aim of this study is to establish what proportion of patients had a significant reduction in haemoglobin requiring transfusion thus assessing the necessity of routine post-operative haemoglobin assessment in an inpatient and outpatient patient cohort undergoing TJA.

## 2 Materials and methods

Institutional Review Board approval was provided and we performed a review of prospectively collected data across a database of all primary elective hip and knee TJA cases performed by a single surgeon at an urban tertiary national referral centre from January 2018 through August 2023. Patients that underwent unicompartmental knee arthroplasty or revision arthroplasty were excluded.

All patients meeting inclusion criteria attended anaesthetic led pre-assessment clinics within 3 months of surgery where full blood count, renal and coagulation profiles were assessed. A clinical examination was performed and additional investigations organised as required to ensure optimization prior to surgery. If haemoglobin was found to be below 11 g/dL then they were treated with iron and vitamin B12 until they reached a Hb of >11 g/dL.

Criteria for day-case surgery was in line with previously published standards^18^ and in order to be considered suitable patients must have been ASA grade I or II, without cognitive impairment; adequate overnight support at home; primary surgery (not revision); no narcotic sensitivity/dependency; low falls risk; no past history of Parkinsonism or cerebrovascular event; actively engage in the day-case pathway and ideally live within 90 min of the hospital.

Demographics recorded included age, sex, length of stay, pre-operative and post-operative haemoglobin level, estimated blood loss, length of surgery, smoking status and co-morbidities. Peri-operative morbidities were also recorded.

All patients underwent regional and spinal hypotensive anaesthesia, received intravenous fluids, pre-operative infusion of 1g tranexamic acid followed by a second dose post-operatively. Total hip arthroplasty (THA) was performed by a single fellowship trained consultant surgeon using a standard posterior approach and total knee arthroplasty (TKA) was performed using a standard medial parapatellar approach with a tourniquet and measured resection of the femur and tibia along with use of a diathermy to aid haemostasis. Surgical drains were not used routinely. Blood loss was calculated at wound closure as the volume of liquid in the suction device minus the volume of irrigation fluid used, whilst all swabs were weighed. Any patient undergoing haemoglobin assessment had their blood drawn on day one and day two postoperatively. All patients were followed as per the national joint register protocol. Using haemoglobin values of <8 g/dL in patients without, and <9 g/dL in patients with cardiorespiratory disease as the threshold values triggering transfusion, we assessed pre-operative and post-operative variables amongst patients to identify predictors for transfusion following surgery.

### 2.1 Statistical analysis

All statistics were performed using Stata release 17 (Stata Corp LLC, Texas, USA). Descriptive statistics are reported as means with standard deviations (SD) and 95 % confidence interval (CI) or range. LASSO method was used to ensure that only the essential set of influential variables were included for regression analysis: Age, preoperative haemoglobin level, intraoperative blood loss, arthroplasty type, operative time, gender, co-morbid disease including Cardiovascular, Respiratory, Haematological, Renal, Endocrine (not diabetes), Liver disease, Diabetes, Obesity and Smoking history.

A multivariate logistic regression model was used to identify variables independently associated with the development of postoperative haemoglobin of <8 g/dL and <9 g/dL.

The preoperative haemoglobin value that was most strongly associated with the development of postoperative haemoglobin of <8 g/dL was calculated separately for men and women. A receiver operating characteristic (ROC) curve analysis was performed, and the area under the curve (AUC) was calculated. The optimal cutoff value was determined by the highest value of Youden index, which is calculated to maximize sensitivity and specificity (sensitivity 1 specificity – 1 = Youden index). A p-value of <0.05 was considered statistically significant.

## 3 Results

Four-hundred-ninety elective primary hip or knee TJA procedures were performed within the six year period (Table 1, Table 2 demographics). The mean drop in haemoglobin was 2.1 g/dL (SD 1.0, Range −5.6 to +1) post-operatively. Four patients (0.8 %) had a haemoglobin drop below 8 g/dL and 19 (3.9 %) had a level below 9 g/dL, however only 4 patients (0.8 %) required allogenic blood transfusion. One hundred and six patients (21.9 %) underwent day-case arthroplasty of which there was no re-admissions or complications within 90 days of surgery. None of these day-case TJAs underwent a post-operative haemoglobin assessment, and no patients re-attended with symptomatic anaemia. Gender and surgery type as well as further demographic and preoperative characteristics are summarised in Table 1, Table 2 respectively.

**Table 1:** Demographics of all participants.

Procedure	M/F	Total
**Total knee arthroplasty**	54/73	127
**Total hip arthroplasty**	181/182	363
**Total**	235/255	490

Variable	Mean	Min	Max
	(std dev)		
**Age**	64 (13.4)	16	89
**Hospital length of Stay (days)**	2.5 (2.7)	0	27
**Pre-operative**	13.4 (1.4)	9.4	17.1
**Haemoglobin level (**g/dL)			
**Post-operative**	11.2 (1.4)	6	15.7
**Haemoglobin level (**g/dL)			
**Decrease in Haemoglobin level (**g/dL)	2.1 (1)	−5.6	1
**Intraoperative Blood loss (ml)**	207.2 (156.1)	0	910
**Operative time (mins)**	55.3 (15.6)	12	150

**Table 2:** Logistic regression of factors associated with Postoperative Haemoglobin below 8 g/dl

Postoperative Haemoglobin level below 8 (g/dl)	Coefficient	Std. err.	z	P-value	[95 % conf.	interval
Pre-operative Hb level (g/dl)	−1.82	0.93	−1.96	0.05	−3.64	0
Total Hip replacement	−1.19	1.46	−0.81	0.42	−4.04	1.67
Cardiovascular disease	−1.87	1.45	−1.29	0.2	−4.7	0.97
Renal disease	4.12	1.88	2.19	0.03	0.43	7.81
Haematological disease	1.9	1.41	1.35	0.18	−0.86	4.67
_cons	16.32	9.28	1.76	0.08	−1.87	34.51

### 3.1 Hip arthroplasty

Three hundred sixty-three THAs were performed at a mean age of 62 years (SD 13.9, Range 16–88). The average length of stay for THAs was 2.1 days (SD 2.7, Range 0–27) with 105 day-case hips performed. The mean pre-operative Hb was 13.5 g/dL (SD 1.3, Range 9.6–17.1) with a mean post-operative drop of 2.0 g/dL (SD 1.0, Range −5.6 to +1). The mean estimated blood loss was 251 ml (SD 149, Range 0–910) with a mean operative time of 55 min (SD 15.5, Range 17–150). One patient had a Hb drop to below 8 g/dL whilst 10 patients sustained a drop below 9 g/dL. One patient underwent transfusion following THA due to a combination of low Hb and Chronic kidney disease.

The mean pre-operative Hb in the day-case cohort was 13.9 g/dL compared to 13.2 g/dL in the inpatient group. The mean age at surgery also differed amongst the inpatient and day-case cohort (66.3 v 56 years respectively).

### 3.2 Knee arthroplasty

One hundred twenty-seven TKAs were performed at a mean age of 69.5 years (SD 9.8, Range 31–89). The average length of stay was 3.5 days (SD 2.5, Range 0–15) with 1 day-case total knee performed. The mean pre-operative Hb 13.0 g/dL(SD 1.6, Range 9.4–16.8) with a mean post-operative drop of 2.1 g/dL (SD 1.0, Range −4.5 to +0.3). The mean estimated blood loss was 68 ml (SD 76, Range 0–403) with a mean operative time of 58 min(SD 15.7, Range 12–120). Three patients had a Hb drop to below 8 g/dL whilst 9 patients sustained a drop below 9 g/dL. Three patients underwent transfusion following TKA due to low Hb with comorbidities (hypertension x3, ischaemic heart disease ×1 and CKD x2).

### 3.3 Postoperative outcomes

Multivariate logistic regression indicated that lower pre-operative haemoglobin, higher intraoperative blood loss and co-morbid renal disease were significant factors associated with postoperative haemoglobin level below 8 g/dL and 9 g/dL across all participants. Detailed summaries in Table 3, Table 4 below.

**Table 3:** Logistic regression of factors associated with Postoperative Haemoglobin below 9 g/dl

Postoperative Haemoglobin level below 9 (g/dl)	Coefficient	Std. err.	z	P-value	[95 % conf.	interval]
Pre-operative	−1.57	0.31	−5.04	0	−2.17	−0.96
Hb level (g/dl)						
Blood loss (ml)	0	0	2.5	0.01	0	0.01
Renal disease	0.65	0.73	0.89	0.37	−0.78	2.08
Haematological disease	0.26	0.75	0.35	0.73	−1.2	1.73
Endocrine disease	−1.51	1.24	−1.22	0.22	−3.93	0.92
_cons	15.48	3.53	4.38	0	8.56	22.41

**Table 4:** Results of Multiple linear regression of factors associated with change in Haemoglobin levels (g/dl).

Hb change preoperative to postoperative	Coefficient	Std. err.	t	P > t	[95 % conf.	interval]
Pre-operative	−0.23	0.04	−6.1	0	−0.3	−0.16
Haemoglobin level (g/dl)						
Surgical time (mins)	−0.01	0	−2.7	0.01	−0.02	0
_cons	1.51	0.52	2.93	0	0.5	2.53

Of the patients with a haemoglobin drop below 9 g/dL there were multiple comorbidities (hypertension x11, hyperlipidemia x4, ischaemic heart disease ×2 and cardiac failure x2, CKD x3). On regression analysis co-morbid renal disease was the only significant factor associated with postoperative haemoglobin level below 8 g/dL (4.12; p = 0.03).

Further analysis indicated the following: Lower pre-operative haemoglobin levels and longer surgical times were associated with larger drops in haemoglobin levels postoperatively. Longer operative times were associated with larger intraoperative blood loss while TKA and increasing age were associated with lower intraoperative blood loss when controlled for other factors.

Factors associated with longer inpatient stay were increasing age, lower pre-operative haemoglobin levels, longer operative time and co-morbid liver, renal and respiratory disease. Conversely, THA was associated with shorter inpatient hospital stays when controlled for other factors. See Table 3, Table 4, Table 5, Table 6.

**Table 5:** Multiple linear regression of factors associated with intraoperative blood loss(ml).

Blood loss(ml)	Coefficient	Std. err.	t	P-value	[95 % conf.	interval]
Age (years)	−1.67	0.45	−3.7	0	−2.55	−0.78
Operative time (mins)	3.02	0.37	8.1	0	2.29	3.75
Total Knee replacement	−174.77	14.11	−12	0	−202.49	−147.05
Diabetes	−26.04	19.29	−1.4	0.18	−63.93	11.86
Rheumatological disease	−32.99	21.93	−1.5	0.13	−76.08	10.11
Obesity	23.48	16.94	1.4	0.17	−9.81	56.77
_cons	190.99	34.15	5.6	0	123.89	258.08

**Table 6:** Multiple linear regression of factors associated with hospital stay in days.

Length of inpatient stay (days)	Coefficient	Std. err.	t	P > t	[95 % conf.	interval]
Age	0.04	0.01	4.77	0	0.03	0.06
Pre-operative	−0.17	0.09	−2.04	0.04	−0.34	−0.01
Haemoglobin level (g/dl)						
Blood loss (ml)	0	0	1.44	0.15	0	0
Operative time (mins)	0.02	0.01	2.45	0.02	0	0.03
Total Hip replacement	−0.91	0.32	−2.85	0.01	−1.54	−0.28
Respiratory disease	0.6	0.3	2.01	0.05	0.01	1.18
Renal disease	1.78	0.5	3.57	0	0.8	2.75
Haematological disease	0.34	0.41	0.83	0.41	−0.46	1.14
Endocrine disease	0.32	0.36	0.9	0.37	−0.38	1.02
Rheumatological disease	0.36	0.43	0.84	0.4	−0.48	1.21
Liver disease	2.39	0.71	3.34	0	0.99	3.79
Obesity	0.55	0.33	1.66	0.1	−0.1	1.21
_cons	0.85	1.4	0.61	0.55	−1.91	3.61

## 4 Discussion

Transfusion risk factors have been extensively studied and our findings correlate with previous reports.^19^ In this series TJA was associated with <1 % transfusion rate. The mean estimated blood loss for TKA was 68 ml v 251 ml in the THA group however the mean haemoglobin drop was the same between groups. Perhaps this can be explained by overzealous perioperative fluid administration, syndrome of inappropriate antidiuretic hormone or serum sampling too early following surgery. Regression analysis found that lower pre-operative haemoglobin levels, longer surgery duration, higher blood loss during surgery and renal disease were associated with a haemoglobin drop below 9g dL.

Routine serum sampling is expensive, invasive, painful and poses infection risk. It requires an inpatient stay in someone who otherwise may have undergone day-case surgery. There is manpower required to draw samples and deliver to the lab. It is necessary to monitor haemoglobin in those with risk factors such as cardiac or renal comorbidities. A preoperative haemoglobin <11 g/dL, omitting tranexamic acid, excessive intraoperative blood loss, prolonged surgery time, anaemia symptoms, deranged vitals as well as wound ooze/haematoma are all scenarios where it would be recommended to assess haemoglobin following TJA surgery.

At three months there were no reported complications in the day-case cohort, and we believe this timepoint is sufficient for symptomatic anaemia to declare itself clinically. The World Health Organization defines anaemia as a haemoglobin level below 12 g/dL in females and 13 g/dL in males.^20^ Chaudhry et al. set the threshold haemoglobin <8 g/dL to trigger transfusion and found a lower preoperative haemoglobin, hip arthroplasty and longer operative time independently increased the odds of a drop <8 g/dL, whilst a higher BMI as well as Tranexamic acid independently decreased the odds.^21^ They recommended a preoperative haemoglobin cut off of 12.4 g/dL in females and 13.4 g/dL in males as predictive of a postoperative level of 8 g/dL^1^. Whilst we did not collect sufficient BMI data for analysis at the time of pre-assessment, it was recorded if the patient had a history of obesity. Of the 50 obese patients, the mean post-operative haemoglobin was 11.2 g/dL and only one of these patients had a haemoglobin drop below 9 g/dL.

NICE guidelines recommend a threshold of <7 g/dL in patients without comorbidities and <8 g/dL in those with cardiorespiratory disease.^22^ By instead using a conservative threshold of <8 g/dL and 9 g/dL respectively we feel this is safer and in line with prior publications such as Chaudhry et al. who reported Hb levels <8 g/dL were associated with a higher incidence of AKI, hospital visits and lengthier inpatient stays.^21^ Kildow et al. mirrored these findings with pre-operative Hb below 11.9 correlating with transfusion after surgery.^23^

A 2024 review by Howgate et al. replicated our findings reporting transfusion rates as low as 0.8 % in 367 patients.^24^ Full blood count assessment was performed in most of their patients but it did not influence clinical management. Similarly Gilde et al. reported a 0.6 % transfusion rate in a similar cohort.^25^

A 2018 paper looking at routine complete blood counts following TJA reported a 15.3 % transfusion rate and those who were transfused had a significantly lower preoperative haemoglobin compared to patients who weren't posing the question whether we should only be routinely checking blood counts post-operatively in those with a lower pre-operative haemoglobin value.^23^ Patients in the same study who did not receive TXA were 3.7 times more likely to receive a transfusion.^23^ In line with our findings they recommended that routine postoperative full blood count assessment in patients with a normal preoperative haemoglobin who receive tranexamic acid do not contribute to actionable information.^23^

Hennessy reported a lower transfusion rate of 3.7 % and recommended full blood counts on the first post-operative day offer no clinical benefit as none of their patients required transfusion within the first 24 h and posed a strong argument for elimination of routine haemoglobin checks following elective TJA.^26^ This correlated to other large centre studies^27^ where those with a haemoglobin drop had historical or clinical reasons to perform full blood counts, indicating that routine postoperative checks may no longer be warranted.^26^

Interestingly Wu et al. found in their cohort of 395 patients undergoing elective THA that 77 % had post-operative anaemia by definition, but only 7 % warranted clinical intervention.

It is reasonable to be more vigilant in postoperative thresholds for assessing haemoglobin following total hip arthroplasty as transfusion rates are higher when compared with knee arthroplasty.^28^ The literature is harmonious in its finding that low preoperative haemoglobin levels are a consistent indicator of the need for postoperative transfusion.^15^, ^29^ Yeh et al. recommend a preoperative haemoglobin of 12.4 g/dL or greater in 70- year-olds and older, with 12.1 g/dL being appropriate in those younger than 70.^30^ A lower transfusion rate than the accepted international standard has been attributed to improved perioperative protocols, patient preoptimization, routine use of tranexamic acid, tourniquets (in total knee arthroplasty) and decreased operative times.^31^, ^32^, ^33^, ^34^ Another study assessed the prognostic value of routine haemoglobin testing and concluded that it may not be indicated for knee TJA provided clinicians accept a 0.1 % risk of patients developing severe undiagnosed anaemia (using a threshold of <70 g/L) but more caution should be employed around hip TJA based on risk thresholds.^35^ Previous studies have reported on the combined effect of pre-, intra- and post-operative measures that limit blood loss in arthroplasty patients. Our transfusion rate was the same as other papers where the authors went on to state that routine haemoglobin sampling is unnecessary^24^ and this paper has validated their stance. We believe the combined effect of hypotensive anaesthesia, the use of TXA as well as an experienced arthroplasty surgeon all had a significant role in our low transfusion rate.

With current public health service economics seen around the world, the financial ramifications of routine blood testing without indications should not be overlooked. This compels us to assess the necessity of routine laboratory tests. Other studies looking at routine laboratory tests reported a single full blood count to cost approximately $10/€8.^36^ Whilst individual test costs are modest, the cumulative expense on a national or global level over time is significant. However we do acknowledge this is offset by potential hidden costs of complications arising from omitting routine blood tests but in our experience no patients re-presented to hospital with anaemia related complications therefore reinforcing our stance that the routine monitoring of haemoglobin following TJA does not lead to actionable information.

According to the National Joint Registry report,^37^ there were 105,798 primary hip and 103,303 knee TJA procedures performed across the NHS and independent hospitals. This equates to 209,101 primary TJA procedures performed annually in the United Kingdom. If routine haemoglobin assessment is abandoned this would lead to savings of €1,672,808 per annum which cannot be overlooked for a procedure that does not lead to actionable information.

In order to attempt to minimize unnecessary expenditure, Gbejuade et al. proposed a criteria for requesting postoperative blood tests including preoperative haemoglobin <11 g/dL, cardiac disease, surgical blood loss of >500 ml as well as clinical symptoms and signs of anaemia.^38^ We also recommend that patients with pre-operative levels >11 g/dL may not require routine post-operative full blood count as mean drop in haemoglobin of less than 3 g/dL can be expected.

We found that routine haemoglobin assessment did not lead to intervention and was largely unnecessary in a defined cohort of patients. We demonstrated a mean haemoglobin drop of 2.1 g/dL with less than 1 % requiring transfusion. Over 20 % of our cohort underwent day-case TJA of which there were no re-admissions or adverse effects from not undergoing haemoglobin assessment post-operatively. An adverse effect was deemed to be signs or symptoms attributed to anaemia such as tachycardia, hypotension, dyspnoea or dizziness.

Routine monitoring of haemoglobin should be in at-risk patients principally where Hb < 11 g/dL or those with significant cardiorespiratory or renal disease.

Limitations of our study include its retrospective nature, albeit of prospective data. A single surgeon cohort results may not be applicable to institutions where trainees are carrying out procedures however we believe this standardizes practise and limits variables or confounders in our study. The posterior approach as employed by the senior author is the most common surgical approach used worldwide^39^ for THA and renders our results applicable to surgeons of different experience levels. It is worth mentioning that the literature is contradictory in its findings regarding conventional versus minimally invasive approaches and their effect on patient outcomes.^40^, ^41^

We also did not include body mass index as a risk factor for excessive blood loss as we did not have sufficient data for analysis and our practise has now changed so that we routinely record BMI data at pre-assessment.

## 5 Conclusion

The routine monitoring of haemoglobin in TJA is a costly unnecessary endeavour and does not contribute to actionable information. We recommend that patients with a pre-operative level greater than 11 g/dL may not require routine post-operative full blood count as mean drop in haemoglobin of less than 3 g/dL can be expected. We continue to advocate that day-case TJA is safe in appropriately selected patients.

## CRediT authorship contribution statement

**David Moore:** Corresponding author): first author; data collection & interpretation, manuscript preparation. **Henry Turner:** MB BCh BAO, MRCS, Formal analysis. **Jess Rotaru:** MB BCh BAO: data collection. **Ciara Doran:** CRediT - research concept, ethics application. **James Cashman:** FRCS: senior author, Writing – original draft.

## Ethical clearance statement

Ethical approval from the authors institution was provided by the institutional review board at our institution in May 2023.

## Declarations

Ethics approval and consent to participate: Ethical approval from the authors institution was provided by the institutional review board.

## Consent for publication

Not applicable.

## Availability of data and materials

The datasets used and/or analysed during the current study are available from the corresponding author on reasonable request.

## Ethical clearance was provided by the IRB in cappagh hospital

Informed Consent has been obtained from patient or guardian for the study's participation and publication.

## Funding

This research did not receive any specific grant from funding agencies in the public, commercial or not-for-profit sectors.

## Declaration of competing interest

The authors declare that they have no competing interests.
